# 4,6-Bis(4-fluoro­phen­yl)-2-phenyl-1*H*-indazol-3(2*H*)-one

**DOI:** 10.1107/S1600536811016369

**Published:** 2011-05-07

**Authors:** R. J. Butcher, M. Akkurt, S. Samshuddin, B. Narayana, H. S. Yathirajan

**Affiliations:** a Department of Chemistry, Howard University, 525 College Street NW, Washington, DC 20059, USA; b Department of Physics, Faculty of Sciences, Erciyes University, 38039 Kayseri, Turkey; c Department of Studies in Chemistry, Mangalore University, Mangalagangotri 574 199, India; d Department of Studies in Chemistry, University of Mysore, Manasagangotri, Mysore 570 006, India

## Abstract

In the title compound, C_25_H_16_F_2_N_2_O, the pyrazole ring is almost planar (r.m.s. deviation = 0.028 Å) and makes a dihedral angle of 5.86 (11)° with the indazole benzene ring. The dihedral angle between the pyrazole ring and the unsubstituted phenyl ring is 28.19 (11)°. The dihedral angles between the unsubstituted phenyl and the two fluoro­phenyl groups are 57.69 (10) and 18.01 (10)°. In the crystal, mol­ecules are linked by inter­molecular N—H⋯O and C—H⋯F inter­actions, forming infinite chains along the *b* axis with graph-set motif *R*_3_^2^(19). The crystal structure is further consolidated by π–π stacking [centroid–centroid distances = 3.5916 (13) and 3.6890 (13) Å] and C—H⋯π inter­actions.

## Related literature

For the pharmacological activity of indazole derivatives, see: Beylin *et al.* (1991 ▶); George *et al.* (1998 ▶); Jain *et al.* (1987 ▶); Palazzo *et al.* (1966 ▶); Popat *et al.* (2003 ▶); Roman (1990 ▶). For related structures, see: van der Helm *et al.* (1979 ▶); Fun *et al.* (2010 ▶). For hybridization and electron delocalization around N atoms, see: Susindran *et al.* (2010 ▶); Jin *et al.* (2004 ▶). For graph-set analysis, see: Etter (1990 ▶); Bernstein *et al.* (1995 ▶).
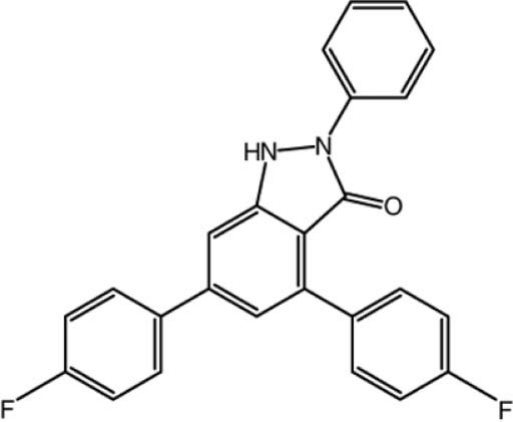


## Experimental

### 

#### Crystal data

C_25_H_16_F_2_N_2_O
*M* *_r_* = 398.40
Orthorhombic, 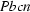

*a* = 15.2947 (4) Å
*b* = 11.6259 (2) Å
*c* = 20.9388 (5) Å
*V* = 3723.23 (15) Å^3^
*Z* = 8
Mo *K*α radiation
μ = 0.10 mm^−1^
*T* = 123 K
0.49 × 0.38 × 0.23 mm

#### Data collection

Oxford Diffraction Xcalibur Ruby Gemini diffractometer
Absorption correction: multi-scan (*CrysAlis PRO*; Oxford Diffraction, 2007 ▶) *T*_min_ = 0.895, *T*_max_ = 0.977
19870 measured reflections
3827 independent reflections
3416 reflections with *I* > 2σ(*I*)
*R*_int_ = 0.029

#### Refinement

*R*[*F* ^2^ > 2σ(*F* ^2^)] = 0.057
*wR*(*F* ^2^) = 0.135
*S* = 1.09
3827 reflections
275 parameters
1 restraint
H atoms treated by a mixture of independent and constrained refinement
Δρ_max_ = 0.65 e Å^−3^
Δρ_min_ = −0.35 e Å^−3^

### 

Data collection: *CrysAlis PRO* (Oxford Diffraction, 2007 ▶); cell refinement: *CrysAlis PRO*; data reduction: *CrysAlis RED* (Oxford Diffraction, 2007 ▶); program(s) used to solve structure: *SIR97* (Altomare *et al.*, 1999 ▶); program(s) used to refine structure: *SHELXL97* (Sheldrick, 2008 ▶); molecular graphics: *ORTEP-3 for Windows* (Farrugia, 1997 ▶); software used to prepare material for publication: *WinGX* (Farrugia, 1999 ▶) and *PLATON* (Spek, 2009 ▶).

## Supplementary Material

Crystal structure: contains datablocks global, I. DOI: 10.1107/S1600536811016369/qk2004sup1.cif

Structure factors: contains datablocks I. DOI: 10.1107/S1600536811016369/qk2004Isup2.hkl

Supplementary material file. DOI: 10.1107/S1600536811016369/qk2004Isup3.cml

Additional supplementary materials:  crystallographic information; 3D view; checkCIF report

## Figures and Tables

**Table 1:** Hydrogen-bond geometry (Å, °) *Cg*5 is the centroid of the C20–C25 phenyl ring.

*D*—H⋯*A*	*D*—H	H⋯*A*	*D*⋯*A*	*D*—H⋯*A*
N2—H*N*2⋯O1^i^	0.86 (2)	2.00 (2)	2.830 (2)	162 (2)
C6—H6⋯F1^ii^	0.93	2.49	3.362 (2)	156
C15—H15⋯*Cg*5^iii^	0.93	2.85	3.656 (2)	145

Symmetry codes: (i) 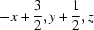; (ii) 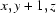; (iii) 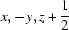.

**Fig. 1.**
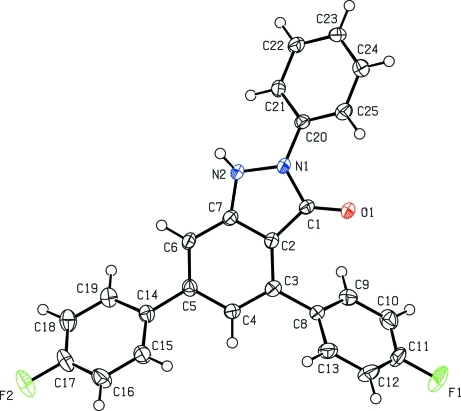
Molecular structure of the title compound showing the atom labeling scheme. Displacement ellipsoids for non-H atoms are drawn at the 50% probability level.

**Fig. 2.**
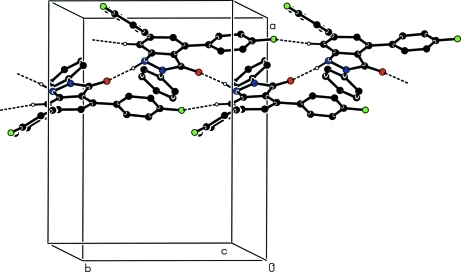
A partial packing diagram of the title structure viewed down the c axis. N—H···O and C—H···F hydrogen bondings (dashed lines) link the molecules, forming R23(19) graph-set motifs along the b axis of the unit cell. H atoms not involved in hydrogen bonds have been omitted for clarity.

## supplementary crystallographic information

### Comment

In last few decades, much attention has been paid to the synthesis of heterocycles containing 1,2-diazole systems like indazole mainly due to their broad spectrum of pharmacological properties. Indazole derivatives possess variety of pharmacological activities such as analgesic, anti inflammatory, antidepressant, antihypertensive, antiviral and anticancer activities (Jain *et al.*, 1987; Palazzo *et al.*, 1966; Popat *et al.*, 2003; Beylin *et al.*, 1991; George *et al.*, 1998; Roman, 1990).

The crystal structure of indazole derivative, *viz*., 1,2,4,5-tetrahydro-7-methoxy-3*H*-benz[*g*]indazol-3-one monohydrate (van der Helm *et al.*, 1979) has been reported. Also the crystal structure of methyl 4,6-bis(4-fluorophenyl)-2-oxocyclohex-3-ene-1-carboxylate, the precursor of the title compound, has been reported (Fun *et al.*, 2010). In view of the importance of indazole derivatives, the title compound (I) is synthesized and its crystal structure is reported here.

The pyrazole ring (N1/N2/C1/C2/C7) in (I), (Fig. 1), is almost planar with the largest deviations from the mean plane being 0.039 (2) Å for C7 and -0.035 (2) Å for N2. The sum of the surrounding angles around N1 in the pyrazole ring is 358.15 (15)°, in accordance with the *sp*^2^ hybridization of the N1 atom (Susindran *et al.*, 2010). The C1—N1 and C7—N2 bond lengths in the pyrazole ring are 1.390 (2) and 1.367 (3) Å, respectively. The values of these distances are shorter than the pertinent single bond length of 1.443 Å and are longer than the double bond length of 1.269 Å (Jin *et al.*, 2004). This case indicates electron delocalization.

The dihedral angle between the pyrazole ring and the indazole benzene ring in (I), (Fig. 1), is 5.86 (11)°. The dihedral angle between the two fluorophenyl groups is 42.56 (11)°, and the dihedral angle between the five-membered pyrazole ring and the unsubstituted phenyl ring is 28.19 (11)°. The unsubstituted phenyl ring and the two fluorophenyl groups make dihedral angles of 57.69 (10) and 18.01 (10)°, respectively, with each other.

In the crystal structure, molecules are linked by intermolecular N—H···O and C—H···F interactions, forming *R*^2^_3_(19) graph-set motifs (Etter, 1990; Bernstein *et al.*, 1995) along the *b* axis of the unit cell (Table 1, Fig. 2). In addition, the crystal structure is consolidated by C—H···π and π-π stacking [*Cg*1···*Cg*3(3/2 - *x*, 1/2 + *y*, *z*) = 3.5916 (13) Å and *Cg*3···*Cg*3(1 - *x*, *y*, 1/2 - *z*) = 3.6890 (13) Å; *Cg*1 and *Cg*3 are the centroids of the N1/N2/C1/C2/C7 pyrazole ring and C8—C13 benzene ring, respectively] interactions.

### Experimental

A mixture of methyl 4,6-bis(4-fluorophenyl)-2-oxocyclohex-3-ene-1-carboxylate (3.42 g, 0.01 mol) and phenyl hydrazine (1.08 g, 0.01 mol) in 50 ml e thanol containing 1 ml glacial acetic acid was refluxed for 10 h. The reaction mixture was cooled and poured into 50 ml ice-cold water. The precipitate was collected by filtration and purified by recrystallization from ethanol. Yellow prisms of (I) were grown from DMF by slow evaporation (m.p.: > 523 K, yield: 58%).

### Refinement

All H atoms attached to C atoms were placed in their calculated positions (aromatic C—H = 0.93 Å) and refined using a riding model with *U*_iso_(H) = 1.2*U*_eq_(C). The N-bound H atom was located from a difference map and refined with a distance restraint N—H = 0.86±0.01 Å, and with *U*_iso_(H) = 1.2*U*_eq_(N).

### Crystal data

**Table:**

C_25_H_16_F_2_N_2_O	*F*(000) = 1648
*M**_r_* = 398.40	*D*_x_ = 1.421 Mg m^−^^3^
Orthorhombic, *P**b**c**n*	Mo *K*α radiation, λ = 0.71073 Å
Hall symbol: -P 2n 2ab	Cell parameters from 11209 reflections
*a* = 15.2947 (4) Å	θ = 5.2–37.5°
*b* = 11.6259 (2) Å	µ = 0.10 mm^−^^1^
*c* = 20.9388 (5) Å	*T* = 123 K
*V* = 3723.23 (15) Å^3^	Prism, colourless
*Z* = 8	0.49 × 0.38 × 0.23 mm

### Data collection

**Table:**

Oxford Diffraction Xcalibur Ruby Gemini diffractometer	3827 independent reflections
Radiation source: Enhance (Mo) X-ray Source	3416 reflections with *I* > 2σ(*I*)
graphite	*R*_int_ = 0.029
Detector resolution: 10.5081 pixels mm^-1^	θ_max_ = 26.5°, θ_min_ = 5.2°
ω scans	*h* = −19→19
Absorption correction: multi-scan (*CrysAlis PRO*; Oxford Diffraction, 2007)	*k* = −14→14
*T*_min_ = 0.895, *T*_max_ = 0.977	*l* = −26→26
19870 measured reflections	

### Refinement

**Table:**

Refinement on *F*^2^	Primary atom site location: structure-invariant direct methods
Least-squares matrix: full	Secondary atom site location: difference Fourier map
*R*[*F*^2^ > 2σ(*F*^2^)] = 0.057	Hydrogen site location: inferred from neighbouring sites
*wR*(*F*^2^) = 0.135	H atoms treated by a mixture of independent and constrained refinement
*S* = 1.09	*w* = 1/[σ^2^(*F*_o_^2^) + (0.0462*P*)^2^ + 4.0317*P*] where *P* = (*F*_o_^2^ + 2*F*_c_^2^)/3
3827 reflections	(Δ/σ)_max_ < 0.001
275 parameters	Δρ_max_ = 0.65 e Å^−^^3^
1 restraint	Δρ_min_ = −0.35 e Å^−^^3^

### Special details

**Table:**

Geometry. Bond distances, angles *etc*. have been calculated using the rounded fractional coordinates. All su's are estimated from the variances of the (full) variance-covariance matrix. The cell e.s.d.'s are taken into account in the estimation of distances, angles and torsion angles
Refinement. Refinement on *F*^2^ for ALL reflections except those flagged by the user for potential systematic errors. Weighted *R*-factors *wR* and all goodnesses of fit *S* are based on *F*^2^, conventional *R*-factors *R* are based on *F*, with *F* set to zero for negative *F*^2^. The observed criterion of *F*^2^ > σ(*F*^2^) is used only for calculating -*R*-factor-obs *etc*. and is not relevant to the choice of reflections for refinement. *R*-factors based on *F*^2^ are statistically about twice as large as those based on *F*, and *R*-factors based on ALL data will be even larger.

### Fractional atomic coordinates and isotropic or equivalent isotropic displacement parameters (Å^2^)

**Table:**

	*x*	*y*	*z*	*U*_iso_*/*U*_eq_	
F1	0.60370 (13)	−0.57691 (11)	0.22016 (9)	0.0656 (6)	
F2	0.48986 (10)	0.29173 (14)	0.53611 (7)	0.0525 (5)	
O1	0.73203 (11)	−0.14990 (12)	0.11749 (7)	0.0351 (5)	
N1	0.72290 (11)	0.04878 (13)	0.11220 (7)	0.0239 (5)	
N2	0.68782 (11)	0.13815 (13)	0.14893 (8)	0.0241 (5)	
C1	0.70924 (15)	−0.05733 (16)	0.14107 (9)	0.0269 (6)	
C2	0.66455 (14)	−0.03003 (16)	0.20027 (9)	0.0272 (6)	
C3	0.63295 (16)	−0.10034 (17)	0.25063 (10)	0.0323 (6)	
C4	0.60213 (13)	−0.04532 (16)	0.30476 (9)	0.0246 (5)	
C5	0.60116 (12)	0.07752 (16)	0.31012 (9)	0.0230 (5)	
C6	0.62766 (13)	0.14448 (16)	0.25935 (9)	0.0248 (6)	
C7	0.65858 (13)	0.08914 (16)	0.20431 (9)	0.0241 (5)	
C8	0.62753 (14)	−0.22733 (16)	0.24316 (9)	0.0257 (6)	
C9	0.58886 (14)	−0.27250 (18)	0.18834 (10)	0.0291 (6)	
C10	0.57996 (15)	−0.3901 (2)	0.18045 (11)	0.0355 (7)	
C11	0.61074 (16)	−0.46066 (17)	0.22766 (12)	0.0382 (7)	
C12	0.64919 (16)	−0.42035 (19)	0.28203 (12)	0.0391 (7)	
C13	0.65705 (15)	−0.30264 (18)	0.28970 (10)	0.0315 (6)	
C14	0.57027 (12)	0.13239 (17)	0.37010 (9)	0.0246 (6)	
C15	0.58997 (14)	0.08541 (18)	0.42973 (10)	0.0302 (6)	
C16	0.56348 (15)	0.1384 (2)	0.48586 (10)	0.0365 (7)	
C17	0.51680 (14)	0.2389 (2)	0.48129 (10)	0.0355 (7)	
C18	0.49559 (14)	0.28874 (19)	0.42389 (11)	0.0338 (6)	
C19	0.52226 (13)	0.23481 (18)	0.36821 (10)	0.0280 (6)	
C20	0.74707 (13)	0.07083 (15)	0.04813 (9)	0.0216 (5)	
C21	0.71378 (14)	0.16740 (16)	0.01763 (9)	0.0257 (6)	
C22	0.73814 (15)	0.19020 (17)	−0.04494 (9)	0.0303 (6)	
C23	0.79396 (15)	0.11777 (17)	−0.07719 (10)	0.0303 (6)	
C24	0.82668 (15)	0.02163 (18)	−0.04644 (10)	0.0320 (6)	
C25	0.80400 (13)	−0.00225 (17)	0.01629 (10)	0.0279 (6)	
HN2	0.7174 (14)	0.2009 (14)	0.1477 (12)	0.038 (7)*	
H4	0.58140	−0.08940	0.33860	0.0300*	
H6	0.62510	0.22430	0.26150	0.0300*	
H9	0.56880	−0.22300	0.15670	0.0350*	
H10	0.55380	−0.42030	0.14400	0.0430*	
H12	0.66960	−0.47070	0.31310	0.0470*	
H13	0.68250	−0.27360	0.32670	0.0380*	
H15	0.62150	0.01710	0.43190	0.0360*	
H16	0.57700	0.10670	0.52540	0.0440*	
H18	0.46410	0.35710	0.42240	0.0400*	
H19	0.50800	0.26720	0.32890	0.0340*	
H21	0.67550	0.21630	0.03890	0.0310*	
H22	0.71650	0.25530	−0.06540	0.0360*	
H23	0.80960	0.13330	−0.11920	0.0360*	
H24	0.86430	−0.02760	−0.06810	0.0380*	
H25	0.82670	−0.06660	0.03680	0.0330*	

### Atomic displacement parameters (Å^2^)

**Table:**

	*U*^11^	*U*^22^	*U*^33^	*U*^12^	*U*^13^	*U*^23^
F1	0.0954 (13)	0.0186 (7)	0.0829 (12)	−0.0145 (7)	0.0149 (10)	−0.0064 (7)
F2	0.0579 (9)	0.0652 (10)	0.0345 (7)	0.0125 (8)	0.0096 (7)	−0.0210 (7)
O1	0.0646 (11)	0.0155 (7)	0.0251 (7)	0.0055 (7)	0.0088 (7)	0.0007 (6)
N1	0.0364 (9)	0.0141 (7)	0.0213 (8)	0.0030 (7)	0.0009 (7)	−0.0001 (6)
N2	0.0366 (9)	0.0142 (7)	0.0216 (8)	0.0004 (7)	0.0023 (7)	−0.0007 (6)
C1	0.0440 (12)	0.0172 (9)	0.0195 (9)	0.0009 (8)	0.0002 (8)	0.0024 (7)
C2	0.0421 (11)	0.0179 (9)	0.0216 (9)	0.0008 (8)	0.0008 (8)	−0.0011 (7)
C3	0.0489 (13)	0.0216 (10)	0.0265 (10)	−0.0039 (9)	0.0052 (9)	0.0002 (8)
C4	0.0311 (10)	0.0216 (9)	0.0210 (9)	−0.0026 (8)	0.0005 (8)	0.0014 (7)
C5	0.0253 (9)	0.0225 (9)	0.0213 (9)	0.0023 (7)	−0.0030 (8)	−0.0027 (7)
C6	0.0344 (11)	0.0149 (8)	0.0250 (10)	0.0026 (7)	−0.0019 (8)	−0.0011 (7)
C7	0.0326 (10)	0.0177 (9)	0.0220 (9)	−0.0008 (8)	−0.0024 (8)	0.0011 (7)
C8	0.0365 (11)	0.0184 (9)	0.0222 (9)	−0.0025 (8)	0.0058 (8)	−0.0002 (7)
C9	0.0345 (11)	0.0292 (10)	0.0237 (10)	0.0015 (9)	0.0011 (9)	0.0034 (8)
C10	0.0384 (12)	0.0360 (12)	0.0321 (11)	−0.0130 (10)	0.0035 (9)	−0.0104 (9)
C11	0.0496 (14)	0.0153 (9)	0.0496 (14)	−0.0070 (9)	0.0130 (11)	−0.0019 (9)
C12	0.0503 (14)	0.0262 (11)	0.0407 (13)	0.0018 (10)	0.0019 (11)	0.0134 (10)
C13	0.0417 (12)	0.0291 (11)	0.0238 (10)	−0.0061 (9)	−0.0023 (9)	0.0046 (8)
C14	0.0247 (9)	0.0246 (10)	0.0244 (10)	−0.0009 (7)	−0.0005 (8)	−0.0038 (8)
C15	0.0351 (11)	0.0303 (10)	0.0253 (10)	0.0051 (9)	0.0007 (9)	−0.0019 (8)
C16	0.0419 (13)	0.0449 (13)	0.0228 (10)	0.0039 (10)	0.0002 (9)	−0.0021 (9)
C17	0.0329 (11)	0.0451 (13)	0.0284 (11)	0.0006 (10)	0.0059 (9)	−0.0150 (10)
C18	0.0283 (10)	0.0330 (11)	0.0400 (12)	0.0068 (9)	0.0025 (9)	−0.0083 (10)
C19	0.0261 (10)	0.0292 (10)	0.0287 (10)	0.0027 (8)	−0.0029 (8)	−0.0039 (8)
C20	0.0276 (9)	0.0180 (9)	0.0191 (9)	−0.0041 (7)	−0.0018 (7)	0.0010 (7)
C21	0.0368 (11)	0.0191 (9)	0.0213 (9)	0.0043 (8)	−0.0013 (8)	−0.0022 (7)
C22	0.0479 (13)	0.0206 (9)	0.0223 (10)	0.0003 (9)	−0.0040 (9)	0.0026 (8)
C23	0.0452 (12)	0.0243 (10)	0.0215 (9)	−0.0072 (9)	0.0054 (9)	0.0021 (8)
C24	0.0370 (11)	0.0273 (11)	0.0318 (11)	0.0024 (9)	0.0095 (9)	−0.0007 (9)
C25	0.0317 (10)	0.0221 (9)	0.0298 (10)	0.0028 (8)	0.0011 (8)	0.0037 (8)

### Geometric parameters (Å, °)

**Table:**

F1—C11	1.365 (2)	C15—C16	1.387 (3)
F2—C17	1.366 (3)	C16—C17	1.373 (3)
O1—C1	1.234 (2)	C17—C18	1.373 (3)
N1—N2	1.400 (2)	C18—C19	1.385 (3)
N1—C1	1.390 (2)	C20—C25	1.387 (3)
N1—C20	1.415 (2)	C20—C21	1.388 (3)
N2—C7	1.367 (3)	C21—C22	1.388 (3)
N2—HN2	0.859 (18)	C22—C23	1.376 (3)
C1—C2	1.451 (3)	C23—C24	1.384 (3)
C2—C3	1.419 (3)	C24—C25	1.387 (3)
C2—C7	1.391 (3)	C4—H4	0.9300
C3—C4	1.384 (3)	C6—H6	0.9300
C3—C8	1.487 (3)	C9—H9	0.9300
C4—C5	1.433 (3)	C10—H10	0.9300
C5—C14	1.486 (3)	C12—H12	0.9300
C5—C6	1.379 (3)	C13—H13	0.9300
C6—C7	1.402 (3)	C15—H15	0.9300
C8—C13	1.386 (3)	C16—H16	0.9300
C8—C9	1.394 (3)	C18—H18	0.9300
C9—C10	1.384 (3)	C19—H19	0.9300
C10—C11	1.368 (3)	C21—H21	0.9300
C11—C12	1.364 (3)	C22—H22	0.9300
C12—C13	1.383 (3)	C23—H23	0.9300
C14—C15	1.396 (3)	C24—H24	0.9300
C14—C19	1.400 (3)	C25—H25	0.9300
			
N2—N1—C1	111.25 (15)	C17—C18—C19	118.4 (2)
N2—N1—C20	119.12 (14)	C14—C19—C18	121.05 (19)
C1—N1—C20	127.78 (15)	N1—C20—C21	119.13 (17)
N1—N2—C7	106.38 (14)	C21—C20—C25	120.29 (18)
C7—N2—HN2	123.5 (17)	N1—C20—C25	120.58 (17)
N1—N2—HN2	114.3 (15)	C20—C21—C22	119.36 (18)
N1—C1—C2	104.38 (15)	C21—C22—C23	120.86 (19)
O1—C1—C2	131.73 (18)	C22—C23—C24	119.36 (19)
O1—C1—N1	123.89 (18)	C23—C24—C25	120.8 (2)
C1—C2—C3	132.02 (18)	C20—C25—C24	119.32 (18)
C1—C2—C7	107.51 (16)	C3—C4—H4	119.00
C3—C2—C7	120.41 (18)	C5—C4—H4	119.00
C2—C3—C4	117.28 (18)	C5—C6—H6	121.00
C4—C3—C8	121.73 (18)	C7—C6—H6	121.00
C2—C3—C8	120.85 (18)	C8—C9—H9	120.00
C3—C4—C5	121.89 (18)	C10—C9—H9	120.00
C4—C5—C6	119.97 (17)	C9—C10—H10	121.00
C4—C5—C14	119.84 (17)	C11—C10—H10	121.00
C6—C5—C14	120.19 (17)	C11—C12—H12	121.00
C5—C6—C7	118.29 (17)	C13—C12—H12	121.00
C2—C7—C6	121.95 (17)	C8—C13—H13	120.00
N2—C7—C6	128.04 (17)	C12—C13—H13	119.00
N2—C7—C2	110.00 (16)	C14—C15—H15	119.00
C3—C8—C13	122.36 (18)	C16—C15—H15	119.00
C9—C8—C13	118.64 (18)	C15—C16—H16	121.00
C3—C8—C9	118.97 (18)	C17—C16—H16	121.00
C8—C9—C10	120.81 (19)	C17—C18—H18	121.00
C9—C10—C11	118.2 (2)	C19—C18—H18	121.00
F1—C11—C10	118.9 (2)	C14—C19—H19	119.00
C10—C11—C12	123.0 (2)	C18—C19—H19	119.00
F1—C11—C12	118.0 (2)	C20—C21—H21	120.00
C11—C12—C13	118.3 (2)	C22—C21—H21	120.00
C8—C13—C12	121.0 (2)	C21—C22—H22	120.00
C15—C14—C19	118.13 (18)	C23—C22—H22	119.00
C5—C14—C15	121.30 (18)	C22—C23—H23	120.00
C5—C14—C19	120.55 (17)	C24—C23—H23	120.00
C14—C15—C16	121.40 (19)	C23—C24—H24	120.00
C15—C16—C17	118.1 (2)	C25—C24—H24	120.00
F2—C17—C18	118.3 (2)	C20—C25—H25	120.00
C16—C17—C18	122.9 (2)	C24—C25—H25	120.00
F2—C17—C16	118.77 (19)		
			
C1—N1—N2—C7	5.3 (2)	C6—C5—C14—C19	35.8 (3)
C20—N1—N2—C7	171.01 (17)	C4—C5—C14—C19	−143.24 (19)
N2—N1—C1—O1	178.1 (2)	C4—C5—C6—C7	−2.7 (3)
C20—N1—C1—O1	14.0 (3)	C4—C5—C14—C15	38.4 (3)
N2—N1—C1—C2	−1.4 (2)	C5—C6—C7—N2	179.9 (2)
C20—N1—C1—C2	−165.57 (19)	C5—C6—C7—C2	−1.2 (3)
C1—N1—C20—C25	−35.8 (3)	C3—C8—C9—C10	−177.9 (2)
N2—N1—C20—C21	−18.2 (3)	C3—C8—C13—C12	178.4 (2)
C1—N1—C20—C21	144.9 (2)	C9—C8—C13—C12	0.5 (3)
N2—N1—C20—C25	161.15 (18)	C13—C8—C9—C10	0.1 (3)
N1—N2—C7—C6	171.78 (19)	C8—C9—C10—C11	−0.5 (3)
N1—N2—C7—C2	−7.2 (2)	C9—C10—C11—F1	−179.0 (2)
N1—C1—C2—C3	−180.0 (2)	C9—C10—C11—C12	0.4 (4)
N1—C1—C2—C7	−3.0 (2)	C10—C11—C12—C13	0.2 (4)
O1—C1—C2—C3	0.5 (4)	F1—C11—C12—C13	179.6 (2)
O1—C1—C2—C7	177.5 (2)	C11—C12—C13—C8	−0.7 (4)
C1—C2—C7—C6	−172.62 (19)	C19—C14—C15—C16	−0.4 (3)
C1—C2—C7—N2	6.4 (2)	C5—C14—C19—C18	−177.86 (19)
C1—C2—C3—C4	172.4 (2)	C5—C14—C15—C16	178.00 (19)
C3—C2—C7—N2	−176.14 (19)	C15—C14—C19—C18	0.5 (3)
C3—C2—C7—C6	4.8 (3)	C14—C15—C16—C17	0.2 (3)
C7—C2—C3—C8	171.4 (2)	C15—C16—C17—F2	179.7 (2)
C1—C2—C3—C8	−11.9 (4)	C15—C16—C17—C18	−0.1 (3)
C7—C2—C3—C4	−4.3 (3)	C16—C17—C18—C19	0.3 (3)
C4—C3—C8—C9	127.1 (2)	F2—C17—C18—C19	−179.50 (19)
C2—C3—C4—C5	0.5 (3)	C17—C18—C19—C14	−0.5 (3)
C4—C3—C8—C13	−50.8 (3)	N1—C20—C21—C22	179.20 (18)
C2—C3—C8—C13	133.7 (2)	C25—C20—C21—C22	−0.1 (3)
C2—C3—C8—C9	−48.4 (3)	N1—C20—C25—C24	−179.86 (18)
C8—C3—C4—C5	−175.18 (19)	C21—C20—C25—C24	−0.6 (3)
C3—C4—C5—C6	3.0 (3)	C20—C21—C22—C23	0.7 (3)
C3—C4—C5—C14	−177.90 (19)	C21—C22—C23—C24	−0.6 (3)
C6—C5—C14—C15	−142.5 (2)	C22—C23—C24—C25	−0.1 (3)
C14—C5—C6—C7	178.28 (17)	C23—C24—C25—C20	0.7 (3)

### Hydrogen-bond geometry (Å, °)

**Table:**

Cg5 is the centroid of the C20–C25 phenyl ring.

**Table:**

*D*—H···*A*	*D*—H	H···*A*	*D*···*A*	*D*—H···*A*
N2—HN2···O1^i^	0.86 (2)	2.00 (2)	2.830 (2)	162 (2)
C6—H6···F1^ii^	0.93	2.49	3.362 (2)	156
C21—H21···N2	0.93	2.48	2.799 (3)	100
C25—H25···O1	0.93	2.43	2.941 (3)	115
C15—H15···Cg5^iii^	0.93	2.85	3.656 (2)	145

Symmetry codes: (i) −*x*+3/2, *y*+1/2, *z*; (ii) *x*, *y*+1, *z*; (iii) *x*, −*y*, *z*+1/2.
